# Thymidine phosphorylase promotes SARS-CoV-2 spike protein-driven lung tumor development

**DOI:** 10.3389/fimmu.2026.1798566

**Published:** 2026-03-31

**Authors:** Cayleigh Wallace, Alex Gileles-Hillel, Amelia Cox, David Gozal, Wei Li, Hong Yue

**Affiliations:** 1 Department of Biomedical Sciences, Joan C. Edwards School of Medicine at Marshall University, Huntington, WV, United States; 2 Pediatric Pulmonology & Sleep, Department of Pediatrics, Hadassah Medical Center, and Faculty of Medicine, The Hebrew University, Jerusalem, Israel; 3 Department of Pediatrics, Joan C. Edwards School of Medicine at Marshall University, Huntington, WV, United States

**Keywords:** lung cancer development, lung injury and fibrosis, SARS-CoV-2 spike protein, stat3, thymidine phosphorylase

## Abstract

**Background:**

COVID-19 survivors exhibit increased interstitial lung fibrosis, a known risk factor for lung cancer. We investigated whether SARS-CoV-2 spike protein (SP)-induced lung injury and elevated thymidine phosphorylase (TYMP) promote lung tumorigenesis.

**Methods:**

A TriNetX retrospective cohort analysis was combined with mechanistic studies in K18-hACE2^TG^ and K18-hACE2^TG^/*Tymp^–/–^* mice. Mice received intratracheal SP or control lysate followed by a urethane-induced lung cancer protocol. Lung injury, inflammation, thrombosis, fibrosis, STAT3 activation, cytokine profiles, and tumor burden were assessed. *In vitro* assays evaluated SP- and RBD-induced ACE2 processing.

**Results:**

Propensity score-matched TriNetX cohorts demonstrated an increased lung cancer risk after COVID-19, particularly among current smokers (n = 166,807; RR 1.22; HR 1.50; P<.001). In mice, SP induced acute lung injury, neutrophil infiltration, and microthrombi, which were reduced in TYMP-deficient mice. SP markedly increased lung tumor incidence and aggressiveness, whereas TYMP deficiency reduced tumor formation from 50% to 18% of lung lobes. SP-induced STAT3 upregulation and collagen deposition were significantly attenuated in K18-hACE2^TG^/*Tymp^–/–^* mice. Cytokine profiling revealed a tumor-promoting, myeloid-dominant inflammatory milieu in K18-hACE2^TG^ mice, in contrast to a T cell-inflamed, anti-tumor profile in K18-hACE2^TG^/*Tymp^–/–^* mice. SP and RBD altered ACE2 processing, generating lower-molecular-weight fragments consistent with enhanced turnover.

**Conclusions:**

SARS-CoV-2 SP drives lung injury, fibrosis, and tumorigenesis through a TYMP-dependent mechanism involving STAT3 signaling and inflammatory microenvironment remodeling. COVID-19 significantly increases lung cancer risk, especially in current smokers. TYMP represents a potential therapeutic target to mitigate long-term pulmonary consequences of COVID-19.

## Introduction

COVID-19, caused by Severe Acute Respiratory Syndrome Coronavirus 2 (SARS-CoV-2) (1), has been resulted in more than 778 million confirmed cases and 7.1 million deaths worldwide as of September 2025. Beyond acute infection, a substantial proportion of survivors develop long-term complications, including interstitial lung fibrosis (2), which is observed in approximately 25% of patients at three months and 14% at one year following infection (3, 4). Because pulmonary fibrosis is a well-established risk factor for lung cancer (5, 6), COVID-19 survivors may represent a population at heightened oncologic risk. Although COVID-19 vaccination has not been associated with increased lung cancer incidence (7, 8), recent studies demonstrated that SARS-CoV-2 infection can awaken dormant lung cancer cells and accelerate metastatic outgrowth (9). However, the molecular drivers linking SARS-CoV-2-induced lung injury to subsequent cancer development remain poorly defined.

Emerging evidence indicates that the SARS-CoV-2 spike protein (SP) is intrinsically pathogenic. Independent of viral replication, SP has been shown to exert pro-inflammatory and pro-thrombotic effects and to promote pro-fibrotic remodeling in the lung (10, 11). Circulating SP has been detected in both infected individuals and vaccine recipients (12, 13), underscoring the importance of determining whether SP directly contributes to persistent lung remodeling and tumor-permissive microenvironmental changes.

Thymidine phosphorylase (TYMP) is a cytoplasmic and nuclear protein with pleiotropic signaling and pro-angiogenic functions (14–16). TYMP expression is elevated in multiple pathological conditions, including atherosclerosis, cancer, and diabetes mellitus, and increased TYMP levels are associated with poor cancer prognosis (14, 17). Notably, TYMP expression is markedly increased in the plasma, neutrophils, macrophages, and lungs of patients with COVID-19 (18–20), where it correlates with thrombotic events, inflammation, and respiratory symptoms. TYMP has also been implicated in fibrotic processes in liver cancer and urinary tract disease (21, 22), and elevated TYMP expression in non-malignant tissues predicts multifocal hepatocellular carcinoma (23). Collectively, these observations raise the possibility that SARS-CoV-2-induced TYMP upregulation contributes to post-COVID lung fibrosis and increase susceptibility to lung cancer.

In this study, using a large human cohort from the TriNetX Research Network and complementary mechanistic mouse models, we tested the hypothesis that TYMP mediates SARS-CoV-2 SP-induced lung injury and tumor promotion. We demonstrate that TYMP amplifies SP-driven inflammation, fibrosis, and immune remodeling, thereby facilitating lung tumor development.

## Materials and methods

### Human study examining the association between SARS-CoV-2 infection and cancer incidence

We conducted a retrospective cohort study using de-identified electronic health records from the TriNetX Research Network. Because only aggregated, anonymized data were analyzed, this study did not meet the definition of human subjects research under U.S. federal regulations and did not require Institutional Review Board approval. Consequently, because the dataset did not contain patient-level details, information regarding the number of vaccine doses received per individual was not available and could not be analyzed as a variable. Patients were stratified by smoking status (current, former, or never smokers) and categorized according to documented SARS-CoV-2 infection and COVID-19 vaccination status. Patients with recorded cancer outcomes prior to the prespecified observation window were excluded.

Two sets of comparisons were performed within each smoking stratum.

1. Primary exposure analysis (COVID-19 vs no COVID-19):

- Current smokers: COVID-19 (largely unvaccinated) vs no COVID-19 (vaccinated)
- Former smokers: COVID-19 (unvaccinated) vs no COVID-19 (vaccinated)
- Never smokers: COVID-19 (vaccinated) vs no COVID-19 (vaccinated).

2. Effect-modification analysis (vaccinated vs unvaccinated among COVID-19-positive patients):

• Current, former, and never smokers analyzed separately.

TriNetX implemented 1:1 propensity score matching (PSM) for each comparison based on age, sex, race, and ethnicity. Covariate balance was assessed using standardized mean differences. The primary outcome was incident lung cancer (ICD-10-CM C34). Secondary outcomes included incident oral cancer and bladder cancer (ICD-10-CM C67).

Absolute risks, risk differences, risk ratios, and odds ratios were calculated. Time−to−event analyses were performed using Kaplan-Meier curves, log−rank tests, and Cox proportional hazards models with verification of proportionality assumptions. Two−sided P values<0.05 were considered statistically significant. All analyses were conducted within the TriNetX platform using matched cohorts with confirmed covariate balance.

### Generation of SARS-CoV-2 spike protein

Crude SP-containing cell lysate (SP) and control lysate from cells transfected with the empty plasmid pcDNA3.1 (P3.1) were prepared as previously reported (10). Lysates were bulk-generated, aliquoted into single-use tubes (500 µg total protein in 50 µL PBS), and stored at -80°C until use.

### Mouse model of lung cancer

K18-hACE2^TG^ mice (Strain: 034860, The Jackson Laboratory) and K18-hACE2^TG^/*Tymp^–/–^* mice (10) were used. Mice were anesthetized with ketamine/xylazine (100/10 mg/kg), and intubated with an 18-gauge catheter, and administered 500 µg SP or P3.1 lysate intratracheally. Mice were then allowed to recover from anesthesia.

Mice were randomly assigned to two groups. In the first group, whole blood was collected from the inferior vena cava 24 hours after treatment using 0.109 M sodium citrate as an anticoagulant. Blood cell counts were measured using a Hemavet 950SF analyzer. Mice were then euthanized, lungs were inflated to 30 cm H_2_O pressure, tracheas were ligated, and lungs were fixed in 10% formalin, paraffin-embedded, and sectioned for histological examination.

In the second group, mice received intraperitoneal injections of urethane (1 g/kg) beginning one day after SP or P3.1 administration, once weekly for eight weeks, followed by a 20-week latency period (24, 25). Whole blood and plasma were collected, and lungs were processed as described above. Tumors were examined both grossly and microscopically.

Mice had ad libitum access to standard laboratory chow and water, and both sexes were included. All procedures were approved by the Institutional Animal Care and Use Committee of Marshall University (#675) and complied with NIH guidelines.

### Effects of SARS-CoV-2 spike protein on ACE2 expression

BEAS-2B cells were treated with P3.1 or SP lysates for 24 hours, lysed, and processed for Western blot analysis of angiotensin-converting enzyme 2 (ACE2), which mediates the entry of SARS-CoV-2 into host cells.

To further assess the effects of SP on ACE2 processing, COS-7 cells, which lack endogenous ACE2 expression, were co-transfected with plasmids encoding human ACE2 and either C9-tagged SP or GFP-tagged S1 receptor-binding domain (RBD). Cell lysates were analyzed by Western blot for ACE2, SP, and RGD, with pan-actin used as a loading control. Antibodies are listed in **Supplementary Table 1**.

### Histology examination

Formalin-fixed, paraffin-embedded lung tissues were sectioned into 6 µm thickness. Hematoxylin and eosin (H&E) and Masson’s Trichrome staining were performed to assess lung architecture, tumor formation, and fibrosis. Single or double immunohistochemistry was conducted using antibodies described in the Results section.

To quantitatively analyze lung injury or fibrosis, images were analyzed using ImageJ, and the data are presented as the percentage of the area covered in each high-power field (HPF). For neutrophil and thrombus quantification, positive cells or thrombi were directly counted in each image with the assistance of ImageJ.

### Mouse plasma cytokine array

Plasma samples (20 µL per mouse) from six randomly selected SP-treated K18-hACE2^TG^ mice and six K18-hACE2^TG^/*Tymp^–/–^* mice were pooled within each group. Cytokine levels were measured using the Proteome Profiler Mouse Cytokine Array Kit (Panel A; R&D Systems). Membranes were scanned, and spot intensities were quantified using ImageJ. Data are presented as log_2_ fold change (K18-hACE2^TG^ vs K18-hACE2^TG^/*Tymp^–/–^*). For cytokines detectable in only one group, a value of one was assigned to enable calculation.

### Statistics

Data were analyzed using GraphPad Prism (version 10.4.2) and are expressed as mean ± SEM. Statistical comparisons were performed using a two-tailed Student’s *t* tests, Mann-Whitney tests, or One- or two-way ANOVA as appropriate. Fisher’s LSD test was used for *post hoc* analysis following two-way ANOVA. A *p* value of ≤ 0.05 was considered statistically significant.

## Results

### Prior SARS-CoV-2 infection is associated with increased lung cancer incidence in humans

Using a retrospective cohort analysis within the TriNetX Research Network, we examined the association between documented SARS-CoV-2 infection and subsequent cancer incidence. Cohort sizes and demographic characteristics after propensity score matching are shown in **Supplementary Table 2**. Prior COVID−19 infection was significantly associated with an increased risk of incident lung cancer, with the highest hazard observed among current smokers, followed by former smokers and never smokers (**Table 1**). In contrast, no consistent increase in risk was observed for oral and bladder cancers (**Table 2**). These findings are consistent with recent reports that SARS-COV-2 infection can promote the outgrowth of dormant lung cancer cells (9) and underscore the need to define the biological mechanisms underlying this association.

**Table 1:** Lung cancer risk and time−to−event results in matched cohorts.

Smoking status & comparison	Risk A	Risk B	RD (A-B)	RR (95% CI)	HR (95% CI)	Log−rank P
Current, COVID−19 vs No COVID−19	1.7%	1.4%	+0.3%	1.22 (1.15–1.29)	1.50 (1.42–1.58)	<.001
Former, COVID−19 vs No COVID−19	1.5%	1.2%	+0.3%	1.24 (1.20–1.29)	1.60 (1.54–1.66)	<.001
Never, COVID−19 vs No COVID−19	0.21%	0.18%	+0.0%	1.16 (1.10–1.21)	1.42 (1.35–1.49)	<.001
Current with COVID−19, Unvaccinated vs Vaccinated	1.9%	1.8%	+0.1%	1.08 (1.03–1.13)	1.02 (0.97–1.07)	.42
Former with COVID−19, Unvaccinated vs Vaccinated	1.5%	1.3%	+0.2%	1.19 (1.15–1.23)	1.14 (1.10–1.17)	<.001

A indicates the exposure cohort listed first in each comparison; B, the comparator. RD, risk difference; RR, risk ratio; HR, hazard ratio. Outcomes exclude individuals with the event before the analysis window. PSM indicates propensity score matching.

**Table 2:** Oral cancer (secondary outcome) in matched cohorts.

Smoking status & comparison	Risk A	Risk B	RD (A–B)	RR (95% CI)	HR (95% CI)	Log−rank P
Current, COVID−19 vs No COVID−19	0.27%	0.29%	−0.0%	0.92 (0.81–1.05)	1.15 (1.01–1.31)	.03
Former, COVID−19 vs No COVID−19	0.28%	0.36%	−0.1%	0.78 (0.72–0.84)	1.01 (0.94–1.09)	.73
Never, COVID−19 vs No COVID−19	0.08%	0.08%	−0.0%	0.97 (0.90–1.04)	1.23 (1.14–1.33)	<.001
Current with COVID−19, Unvaccinated vs Vaccinated	0.28%	0.27%	+0.0%	1.04 (0.92–1.17)	—	.56
Former with COVID−19, Unvaccinated vs Vaccinated	0.27%	0.24%	+0.0%	1.13 (1.05–1.22)	1.09 (1.01–1.18)	.03

A indicates the exposure cohort listed first in each comparison; B, the comparator. RD, risk difference; RR, risk ratio; HR, hazard ratio. Outcomes exclude individuals with the event before the analysis window. PSM indicates propensity score matching.

### Intratracheal delivery of SARS-CoV-2 SP induces lung injury, inflammation, and microthrombus formation

Given that COVID-19 is primarily a respiratory disease, we examined the effect of intratracheal SP administration on lung inflammation and thrombus formation in K18-hACE2^TG^ and K18-hACE2^TG^/*Tymp^–/–^* mice. As shown in **Supplementary Table 3**, intratracheal SP administration did not significantly affect whole blood cell counts at 24 hours.

**Figure 1:**
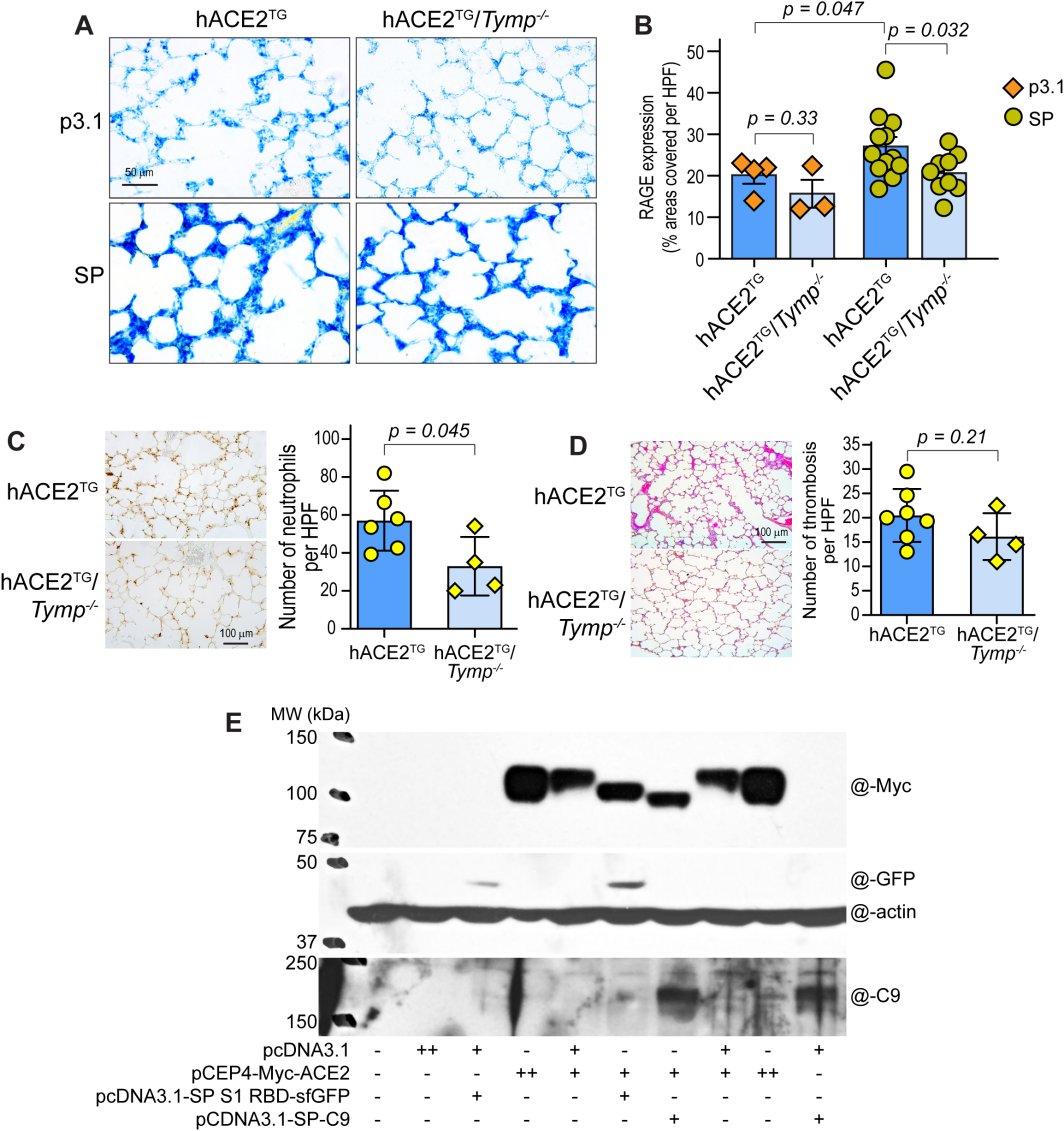
SARS-CoV-2 spike protein (SP) induces lung injury, inflammatory cell infiltration, microthrombus formation, and ACE2 shedding. K18-hACE2^TG^ (hACE2^TG^) and K18-hACE2^TG^/*Tymp^–/–^* (hACE2^TG^/*Tymp^–/–^*) mice received intratracheal administration of 500 µg COS-7 cell lysate containing either SP or control (P3.1). Lungs were harvested 24 hours later for histological analysis. **(A, B)** Lung injury was assessed by immunohistochemical staining of the receptor for advance glycated end products (RAGE), visualized with Vector Blue **(A)**. No counterstaining was performed. Images were analyzed using ImageJ, and data are presented as the percentage of area covered per high-power field (HPF, 40x) **(B)**. **(C)** Immunohistochemical staining for myeloperoxidase (MPO) was performed as a marker of neutrophil infiltration. MPO-positive cells (brown) were quantified using ImageJ. **(D)** Hematoxylin & Eosin staining was used to evaluate lung morphology. Microthrombi were counted visually with the assistant of ImageJ. **(E)** COS-7 cells were co-transfected with the indicated plasmids in various combinations using FuGENE^®^ 6 Transfection Reagent. All plasmids (except pCDNA3.1) were obtained from Addgene. Each “+” represents 500 ng plasmid DNA. Cells were harvested 40 hours after transfection and analyzed by Western blot using the indicated antibodies. Antibody dilutions are listed in **Supplementary Table 1**. Data represents two independent biological replicates.

The receptor for advanced glycated end products (RAGE), expressed by bronchiolar epithelial cells, type II alveolar cells, and alveolar macrophages (26), plays an important role in lung inflammation and is considered a marker of acute lung injury. As shown in **Figures 1A, B**, inhalation of either P3.1- or SP-containing cell lysate induced significant lung injury; however, SP caused more severe injury, which was attenuated by TYMP deficiency.

Acute lung injury is accompanied by an influx of neutrophils into the interstitial and bronchioalveolar space (27). We therefore stained lung neutrophils using myeloperoxidase as a marker. Neutrophil infiltration was significantly reduced in K18-hACE2^TG^/*Tymp^–/–^* mice 24 hours after SP exposure (**Figure 1C**), consistent with the reduced lung injury observed in these mice. In contrast, CD68-positive macrophage levels were similar between P3.1 and SP treatments and between genotypes (**Supplementary Figure 1**).

Microthrombi were detected in the lungs of all mice, including controls receiving P3.1 lysates (data not shown). However, SP inhalation induced larger and more extensive thrombus formation, which was attenuated in TYMP-deficient mice (**Figure 1D**). These findings support a previous study that SARS-CoV-2 SP enhances inflammation and thrombosis (10), contributing to lung injury.

Reportedly, binding of the SARS-CoV-2 SP or its RBD to ACE2 induces ACE2 internalization and degradation, resulting in reduced surface ACE2 expression (28, 29). Although BEAS-2B cells express a low level of ACE2, we did not detect ACE2 protein under our experimental conditions in cells treated with P3.1, SP, or vehicle (data not shown). We therefore co-transfected human ACE2 together with SP or RBD into COS-7 cells to examine their effects on ACE2 protein processing. Unexpectedly, instead of observing uniform ACE2 degradation, we found that co-expression of SP or RBD generated distinct lower-molecular weight Myc-tagged ACE2-immunoreactive bands (**Figure 1E**), with SP producing a further smaller fragment. Because the Myc-tag is positioned at the ACE2 N-terminus, the presence of these smaller Myc-positive fragments suggests that SP and RBD induced ACE2 processing that removes portions of the C-terminal region, consistent with that ACE2 is a target of intramembrane proteolysis of γ-secretase, releasing a soluble ACE2 C-terminal fragment (30).

### Intratracheal delivery of SARS-CoV-2 SP increases lung cancer incidence

**Figure 2:**
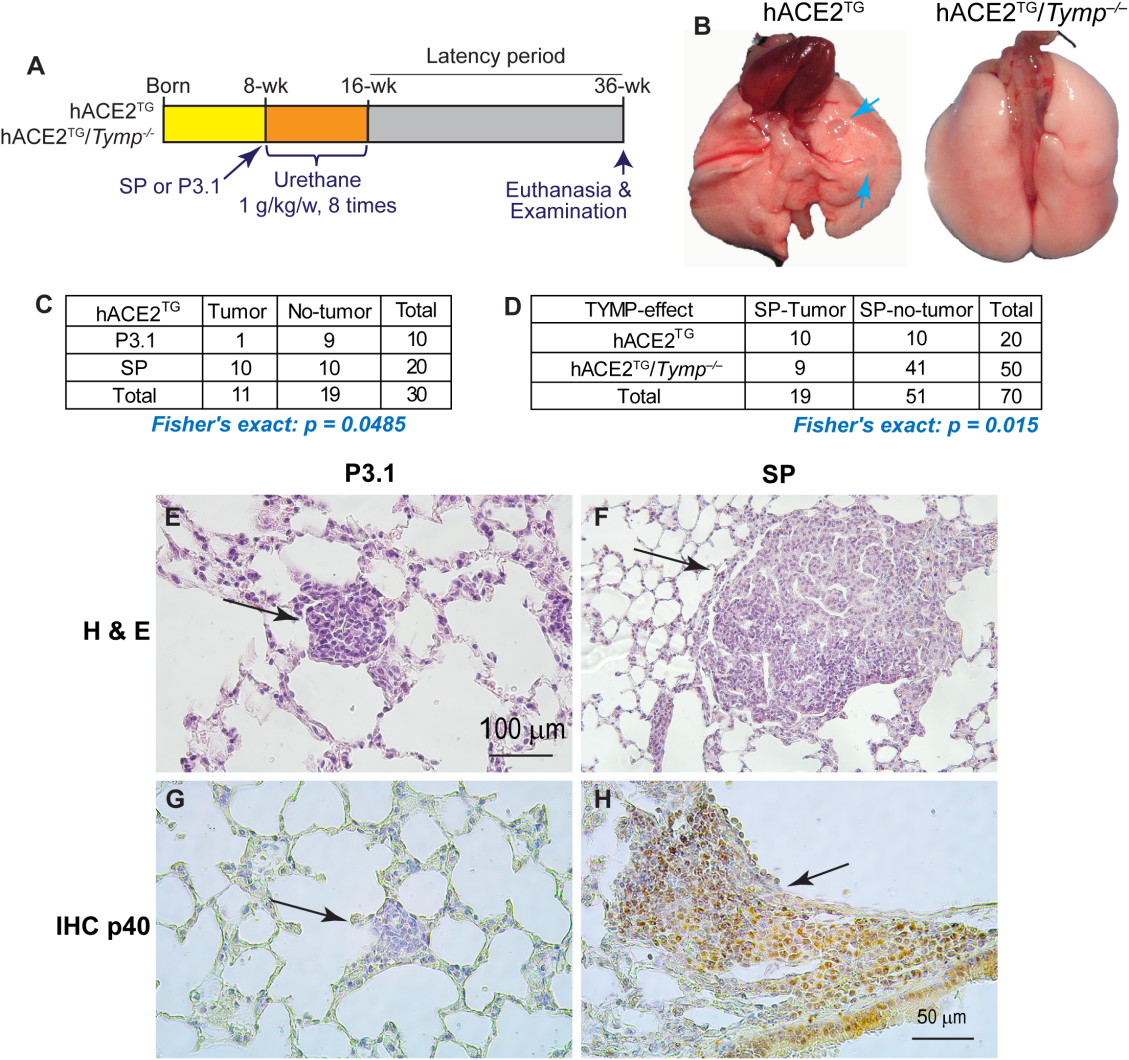
Intratracheal delivery of SARS-CoV-2 Spike protein (SP) increases lung cancer incidence. **(A)** Schematic illustration of the urethane-induced lung cancer model. **(B)** Representative gross images of lung cancer in K18-hACE2^TG^ (hACE2^TG^) mice. The lungs of K18-hACE2^TG^/*Tymp^–/–^* (hACE2^TG^/*Tymp^–/–^*) was shown as control. Blue arrows indicate tumors. **(C)** SP-enhanced lung tumor incidence in hACE2^TG^ mice receiving SP or P3.1 control lysate. **(D)** Comparison of SP-enhanced lung tumor incidence between K18-hACE2hACE2^TG^) and K18-hACE2^TG^/*Tymp^–/–^* (hACE2^TG^/*Tymp^–/–^*) mice. **(E, F)** Hematoxylin & Eosin staining with representative histological images of lung tumors. **(G, H)** Immunohistochemical staining for p40, a marker of lung squamous cell carcinoma. Brown staining indicates p40-positive cells.

Given that lung cancer incidence is significantly increased post-COVID-19 (9), we examined the long-term consequences of SARS-CoV-2 SP-induced lung injury by subjecting mice that received P3.1 or SP treatment to a urethane-induced lung cancer model (**Figure 2A**). P3.1 or SP inhalation, as well as urethane treatment, did not affect mouse body weight among the groups (**Supplementary Figure 2**). No obvious health abnormalities were observed during the latency period. In K18-hACE2^TG^ mice that received SP, tumors (**Figure 2B**) were observed in 10 of 20 lung lobes examined. In contrast, only one tumor was identified among the 10 lobes from mice treated with P3.1 control cell lysate (**Figure 2C****;** Fisher’s exact test-*P* value: 0.0485), indicating that SP exposure significantly promoted lung tumor development. Whereas tumors were detected in 50% of the lobes examined in K18-hACE2^TG^ mice, only nine tumors were found among 50 lobes (18%) in K18-hACE2^TG^/*Tymp^–/–^* mice (**Figure 2D**), indicating that TYMP deficiency markedly reduces SP-induced lung tumorigenesis. Histological analysis revealed that SP-enhanced tumors were more aggressive, as evidenced by their large tumor size (**Figures 2E, F**). Unexpectedly, all tumors found in SP-treated mice were positive for p40 staining (**Figures 2G, H**), a marker of lung squamous cell carcinoma (31, 32), which is not commonly observed in the urethane-induced lung cancer model that typically shows a bronchioloalveolar adenoma phenotype (33).

TYMP inhibits vascular smooth muscle cell (VSMC) proliferation (34, 35); however TYMP deficiency does not affect angiogenesis in mice (36). Using CD31 and α-smooth muscle actin (α-SMA) double immunohistochemistry, we further confirmed that TYMP deficiency also does not affect lung angiogenesis or arteriogenesis in K18-hACE2^TG^ mice (**Supplementary Figure 3**). These findings suggest that the differences in lung cancer incidence observed are not attributable to TYMP’s pro-angiogenic effect or its influence on VSMC function.

### TYMP amplifies STAT3 signaling and drives a profibrotic, protumor microenvironment

**Figure 3:**
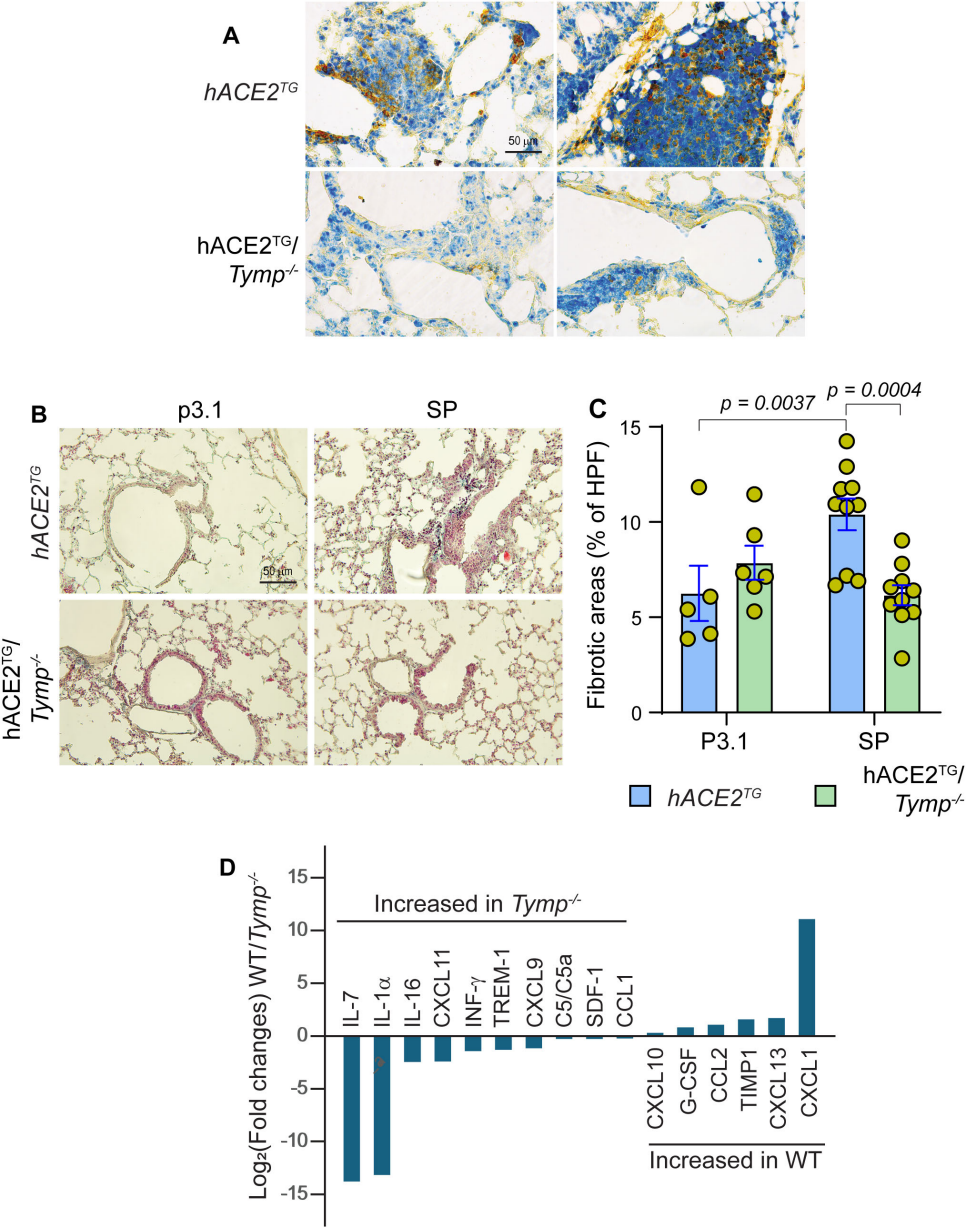
TYMP amplifies STAT3 signaling and drives a profibrotic, pro-tumor microenvironment. **(A)** Lung sections from SP-treated K18-hACE2^TG^ (hACE2^TG^) and K18-hACE2^TG^/*Tymp^–/–^* (hACE2^TG^/*Tymp^–/–^*) mice were double-stained for Y705-phosphorylated STAT3 (Blue) and Total STAT3 (Brown). Two representative images were shown for each genotype. **(B)** & **(C)** Trichrome staining was performed on lung sections from mice subjected to the urethane-induced lung cancer protocol **(B)**. Images were analyzed using ImageJ, and fibrotic areas were quantified by measuring blue-stained regions obtained through color deconvolution. Quantitative data are presented as bar grafts **(C)**. Statistical analyses were performed using Two-way ANOVA followed by Uncorrected Fisher’s LSD *post hoc* testing. **(D)** Cytokine array analysis was performed using plasma pooled from six SP-treated K18-hACE2^TG^ (WT) or K18-hACE2^TG^/*Tymp^–/–^* (*Tymp^–/–^*) mice. Data are presented as Log_2_ fold change of the WT/*Tymp^–/–^* ratio for each cytokines. Only cytokines with changes greater than 10% are shown.

Both RBD and SP increased STAT3 phosphorylation in a TYMP-dependent manner (10). This finding aligns with a previous report that TYMP overexpression enhances STAT3 activation (37). STAT3 has also been implicated in COVID-19 pathology (38). To determine if TYMP-mediated, SP-enhanced STAT3 activation contributes to cancer development, we stained lung tumor sections for Y705-phosphorylated STAT3 and total STAT3. While Y705-STAT3 was widely stained in lung tissues regardless of tumor presence, total STAT3 was predominantly localized within tumors (**Figure 3A**).

Since STAT3 activation promotes fibrosis (39), we assessed lung fibrosis by Trichrome staining (**Figure 3B**). Two-way ANOVA revealed a significant Genotype x Treatment interaction (F(1,27) = 10.67, *p* = 0.003), indicating that the effect of treatment differed by genotype. Neither Genotype (*p* = 0.185) nor Treatment (*p* = 0.150) showed a significant main effect. *Post hoc* comparisons demonstrated that, compared with P3.1-treated controls, SP markedly increased lung fibrosis in K18-hACE2^TG^ mice (**Figure 3C**). In contrast, this SP-induced fibrosis was significantly attenuated in K18-hACE2^TG^/*Tymp^–/–^* mice (difference of differences = −5.848, 95% CI: −9.521 to −2.175).

TYMP deficiency alters the expression of several cytokines (40), which may influence immune disorders. To assess whether the interaction between SP and TYMP modulates cytokine expression, we conducted cytokine array analysis using pooled plasma from SP-treated mice. As shown in **Figure 3D**, **Supplementary Figure 4**, compared with K18-hACE2^TG^, K18-hACE2^TG^/*Tymp^–/–^* mice exhibited increased levels of Th1-associated mediators (IFN-γ, IL-7), CXCR3 ligands (CXCL9, CXCL11), and T-cell-attracting chemokines (IL-16, CCL1), consistent with a T-cell-inflamed, anti-tumor microenvironment. In contrast, K18-hACE2^TG^ mice showed elevated G-CSF, CCL2, CXCL1, TIMP-1, and CXCL13, characteristic of a myeloid-dominated, tumor-promoting inflammatory state, aligning with the higher incidence of lung tumors observed in K18-hACE2^TG^ mice.

## Discussion

In this study, we provide convergent human and experimental evidence that SARS-CoV-2 infection increases the risk of lung cancer and identify TYMP as a previously unrecognized molecular driver of SP-induced lung injury, fibrosis, and tumorigenesis. By integrating large-scale clinical data with mechanistic mouse models, we demonstrate that TYMP amplifies SP-triggered inflammation and fibrotic remodeling, facilitates STAT3 expression, and shapes a tumor-permissive immune microenvironment. These findings establish TYMP as a central pathogenic mediator linking SARS-CoV-2 respiratory injury to increased lung cancer risk.

Our analysis of a large, demographically matched cohort from the TriNetX Research Network revealed a significant increase in lung cancer incidence among individuals with prior SARS-CoV-2 infection. This effect was most pronounced in current smokers, followed by former smokers and never smokers, consistent with the concept that preexisting epithelial injury or chronic inflammation sensitizes the lung to SARS-CoV-2-driven oncogenic stimuli (41, 42). This association was not observed for oral or bladder cancers, indicating organ specificity and involvement of lung-specific mechanisms. Interestingly, a previous study reported that TYMP is not expressed in several tongue cancer cell lines, including HSC3 and SCC-9 (14). These findings align with recent reports that SARS-CoV-2 selectively awakens dormant lung cancer cells and promotes rapid tumor progression (9), underscoring the need to define biological processes that couple viral exposure to oncogenesis.

Because SP is the first viral protein to interact with host cells, we investigated whether SP alone is sufficient to recapitulate lung pathological features associated with tumor development. Intratracheal SP administration caused marked acute lung injury, characterized by heightened RAGE staining, neutrophil infiltration, and extensive microthrombus formation. These effects were significantly attenuated in TYMP-deficient mice, indicating that TYMP amplifies SP-triggered inflammatory and thrombotic responses. Although macrophage infiltration did not differ between genotypes, the robust neutrophilic influx in K18-hACE2^TG^ mice highlights TYMP’s selective role in shaping acute inflammatory responses. Neutrophils promote DNA damage, secrete pro-tumorigenic proteases, and generate neutrophil extracellular traps, processes that may contribute to tumor initiation. Thus, the early inflammatory landscape induced by SP and modulated by TYMP may establish conditions that favor malignant transformation.

Using a urethane-induced lung cancer model, we found that prior SP exposure significantly increased tumor incidence and aggressiveness. We used a urethane dose of 1 g/kg, which has been reported to induce lung tumors with a 60-80% incidence (24, 25). However, in K18-hACE2^TG^ mice treated with P3.1, we observed only ~10% tumor incidence. This discrepancy may reflect the inhibitory effects of ACE2 on tumor proliferation, metastasis, invasion, and angiogenesis across multiple cancer types, including lung cancer (43, 44). SP- or RBD-associated ACE2 shedding or degradation may therefore attenuate this protective effect of ACE2.

Remarkably, tumors in SP-treated mice displayed squamous cell carcinoma morphology with strong p40 positivity, a phenotype atypical for urethane-induced adenomas. These findings suggest that SP-TYMP interactions reprogram the epithelial injury-repair pathways toward squamous metaplasia and malignant transformation. TYMP deficiency dramatically reduced tumor number and size, indicating that TYMP is essential for SP-enhanced lung tumorigenesis. This reduction was not attributable to impaired angiogenesis or altered VSMC function, as TYMP-deficient mice showed normal pulmonary vascular architecture. Instead, our findings point to TYMP’s regulatory role in inflammatory signaling and fibrotic remodeling as key determinants of tumor promotion.

STAT3 is a master regulator of inflammation, fibrosis, and cancer progression (39). Roytenberg et al. reported that SP enhances STAT3 Y705 phosphorylation in BEAS-2B cells in a TYMP-dependent manner (10), we now show that phospho-STAT3 is broadly detected across lung tissues, whereas total STAT3, including unphosphorylated STAT3, is primarily enriched within tumors from K18-hACE2^TG^ mice. STAT3 activation promotes fibroblast-to-myofibroblast transition, extracellular matrix deposition, and epithelial plasticity (45). Unphosphorylated STAT3 also exerts pathophysiological functions, including suppression of VSMC proliferation in TYMP-overexpressing cells (37, 46). Together, these findings support a model in which TYMP-mediated STAT3 signaling links SP-induced injury to fibrosis and carcinogenesis. In agreement with this model, we found that SP markedly increased lung collagen deposition and fibrotic remodeling in K18-hACE2^TG^ mice, whereas fibrosis was significantly attenuated in K18-hACE2^TG^/*Tymp^–/–^*mice. These results support a tangible pathogenic framework, which creates a tumor-permissive niche. However, our data do not determine whether SP administration alone is sufficient to induce long-term pulmonary fibrosis independent of tumor induction, which will require dedicated studies in future work.

Cytokine profiling revealed fundamentally different inflammatory landscapes between SP-treated K18-hACE2^TG^ mice and K18-hACE2^TG^/*Tymp^–/–^* mice. SP-exposed K18-hACE2^TG^ mice exhibited elevated G-CSF, CCL2, CXCL1, TIMP-1, and CXCL13, factors associated with myeloid recruitment, angiogenesis, extracellular matrix remodeling, and tumor promotion. In contrast, K18-hACE2^TG^/*Tymp^–/–^* mice showed enhanced Th1 cytokines (IFN-γ, IL-7), CXCR3 ligands (CXCL9, CXCL11), and T-cell-attracting chemokines (IL-16, CCL1), indicative of an inflamed, anti-tumor immune environment. Overexpression of certain forms of IL-1 α has been shown to lead to tumor regression in some experimental models (47). Thus, TYMP not only amplifies SP-induced injury and fibrosis but also orchestrates an immunologic shift toward tumor-supportive inflammation. This dual role, structural and immunologic, may account for the profound reduction in tumor burden observed in TYMP-deficient mice.

Taken together, our findings support a mechanistic model in which SARS-CoV-2 SP initiates lung injury, neutrophilic inflammation, and microthrombosis; induces sustained TYMP expression; and drives STAT3-mediated fibrosis and immune dysregulation. This creates a microenvironment conducive to malignant transformation or expansion of previously dormant tumor cells. Clinically, these results suggest that COVID-19 survivors may benefit from long-term lung cancer surveillance, particularly those who are current and former smokers. TYMP is a compelling therapeutic target. Tipiracil, an FDA-approved TYMP inhibitor known to inhibit thrombosis (48), may warrant investigation for preventing post-COVID fibrosis or mitigating cancer risk. STAT3-directed therapies may be beneficial in patients with persistent post-COVID lung injury.

This study is limited by its retrospective design, which precludes causal inference. Prospective studies are needed to define post-infection cancer risk. The downstream mechanisms of TYMP remain unclear, as does whether vaccination induces transient TYMP elevation. Future single-cell and spatial analyses should assess TYMP-driven epithelial-stromal-immune interactions and therapeutic targeting in fibrosis-associated oncogenesis. Furthermore, another limitation of this study is that the *in vivo* experiments were performed using intratracheal administration of recombinant SARS-CoV-2 spike protein rather than infection with live virus. While this approach allows us to specifically investigate spike-mediated biological effects, it does not fully recapitulate the complexity of SARS-CoV-2 infection, including viral replication and innate immune sensing of viral RNA.

In conclusion, this study reveals TYMP as a key molecular nexus through which SARS-CoV-2 SP promotes lung injury, fibrosis, and cancer development. By identifying TYMP as both a biomarker and effector of SP-induced pathology, our findings open new avenues for therapeutic intervention and highlight the urgent need for cancer risk assessment in individuals recovering from COVID-19.

**Sources of support:** This work was supported, in whole or in part by the following sources: the Marshall University Institute Fund (to WL), the National Institutes of Health R15HL145573 and 1R01HL177493-01A1 (to WL), the West Virginia Clinical and Translational Science Institute-Pop-Up COVID-19 Fund (to WL) supported by the National Institute of General Medical Sciences (U54GM104942), the Cancer Biology Pilot Grant Program (to WL) supported by the West Virginia IDeA Network of Biomedical Research Excellence (WV-INBRE, P20GM103434). The content is solely the responsibility of the authors and does not necessarily represent the official views of the National Institutes of Health.

## Data Availability

The original contributions presented in the study are included in the article/**Supplementary Material**. Further inquiries can be directed to the corresponding author.
